# Effect of External Force on Agency in Physical Human-Machine Interaction

**DOI:** 10.3389/fnhum.2020.00114

**Published:** 2020-05-08

**Authors:** Satoshi Endo, Jakob Fröhner, Selma Musić, Sandra Hirche, Philipp Beckerle

**Affiliations:** ^1^Chair of Information-Oriented Control, Department of Electrical and Computer Engineering, Technical University of Munich, Munich, Germany; ^2^Elastic Lightweight Robotics Group, Department of Electrical Engineering and Information Technology, Robotics Research Institute, Technische Universität Dortmund, Dortmund, Germany; ^3^Institute for Mechatronic Systems, Mechanical Engineering, Technische Universität Darmstadt, Darmstadt, Germany

**Keywords:** human-centered control, shared control, human-robot interaction, autonomy, agency, haptics

## Abstract

In the advent of intelligent robotic tools for physically assisting humans, user experience, and intuitiveness in particular have become important features for control designs. However, existing works predominantly focus on performance-related measures for evaluating control systems as the subjective experience of a user by large cannot be directly observed. In this study, we therefore focus on agency-related interactions between control and embodiment in the context of physical human-machine interaction. By applying an intentional binding paradigm in a virtual, machine-assisted reaching task, we evaluate how the sense of agency of able-bodied humans is modulated by assistive force characteristics of a physically coupled device. In addition to measuring how assistive force profiles influence the sense of agency with intentional binding, we analyzed the sense of agency using a questionnaire. Remarkably, our participants reported to experience stronger agency when being appropriately assisted, although they contributed less to the control task. This is substantiated by the overall consistency of intentional binding results and the self-reported sense of agency. Our results confirm the fundamental feasibility of the sense of agency to objectively evaluate the quality of human-in-the-loop control for assistive technologies. While the underlying mechanisms causing the perceptual bias observed in the intentional binding paradigm are still to be understood, we believe that this study distinctly contributes to demonstrating how the sense of agency characterizes intuitiveness of assistance in physical human-machine interaction.

## 1. Introduction

In the face of growing elderly population, automated assistive technologies such as powered exoskeleton and rehabilitation devices are expected to play a crucial role for meeting societal demands (Beckerle et al., 2017). A large portion of such assistive technologies involve physical human-machine interactions (HMI) in which a robot is physically coupled with a user to (semi-)autonomously guide the motion (e.g., Marchal-Crespo and Reinkensmeyer, 2009). In order to meet user-dependent requirements in guiding behavior, usually explicit control objectives are minimized. Typically, these cost functions are task-oriented and use a set of performance indices such as the muscular effort (Hamaya et al., 2017) or the task completion time (Erdogan and Argall, 2017) to define the utility of the autonomous behavior of a system. As these control schemes commonly focus on the explicit performance of the user, user experience is notecessarily considered, which might have a strong impact on the user acceptance and long-term usage. However, user experience is of high relevance for designing assistive technologies in which the human is at the center of a control loop (Limerick et al., 2014; Beckerle et al., 2017) and methodologies for objective monitoring of user experience will be valuable for advancements of HMI systems.

To this end, concepts from psychological research may provide means of validating human-centered control designs from the users' perspectives. Previous research has indicated the experienced incorporation of an intelligent tool, i.e., embodiment, appears to be a promising quality measure for a semi-autonomously controlled system (Fröhner et al., 2019). As a subcomponent of embodiment (Longo et al., 2008), in particular, the sense of agency (SoA) refers to an inference about authorship of a sensory event and a believe about whether the sensory outcome was caused by the action of oneself. Accordingly, SoA over an intelligent tool seems to be distinctly relevant when assessing shared-autonomy tasks, which we assume to relate directly to intuitiveness. Previous research on SoA suggests that the central nervous system continuously monitors a discrepancy between the intended movement and corresponding sensory feedback (Wolpert et al., 1995) and evaluates whether the observed sensory event was self-induced (Blakemore et al., 2002). As a result, SoA is reduced, for example, when contiguity between the movement and its sensory outcome is temporally or spatially perturbed, even when their action had resulted in causing the event (Sato and Yasuda, 2005; Farrer et al., 2008). In reverse, people may experience agency over an action produced by another individual when the actions of individuals are sufficiently assimilated with own desired outcome (Dewey and Knoblich, 2014). This is an interesting observation as, in shared-autonomy settings, some external autonomous system might be able to form a collective agency when the assisting force approximates the desired state of the user. Considering this, SoA may become a good holistic connotation for quality of a control system. In order to demonstrate the relationship between the SoA and a physically assistive device, therefore, the present study investigates whether SOA is modulated by the quality of external assistive force, by means of adherence to the human task goal.

In previous studies, questionnaires have been used to reflect users' opinions regarding control designs, (e.g., Lopez-Samaniego and Garcia-Zapirain, 2016) as the internal states of the user such as the intuitiveness and usability of the devices cannot directly be measured. However, using questionnaires may be impractical for tuning control parameters, as an explicit survey of subjective opinions can easily be modulated by a variety of factors, and reliable measurement would require impractically many samples. Furthermore, the physical HMI task and administration of the questionnaire has to take place separately, which appears not suitable for online adaptation of control parameters in a fine temporal resolution. One of the most promising methodologies may be the intentional binding effect (IBE) which has been used to empirically study perceptual changes associated with SoA. IBE is a psychological phenomenon in which the temporal perception of two consecutive events being reported as shorter than the real time-lapse if the events are triggered by the participant herself/himself (Haggard et al., 2002). It is considered that coupling of the self-induced motion and its outcome in the conscience experience attracts the temporal experience of the two sensory events, resulting in perception of a self-induced action outcome being perceived as earlier than it physically is. This subjective contraction of time is considered to be an implicit measure of SoA as it reflects the internal representation of self-produced motion. Previously, the IBE paradigm was used to evaluate the attribution of agency in the presence of autonomic assistance over a series of discrete subtasks (Berberian et al., 2012). In their task, the computer and the participants shared subtasks in different degrees to control an emulated aircraft, and IBE was used to show that the degree of task sharing (or autonomy) modulated SoA. Thus, the autonomy delegated subtasks between the human and the machine, but the action performed by the users were always intact and not perturbed by the autonomy. In applications of physical HMI technologies, on the other hand, the external force from an assistive system continuously and directly influences the motion of the user and the autonomy relates not only to the level of involvement, but also to the adherence to the desired outcome of a user. As mentioned above, there are indications that embodiment can serve as a measure in such situations (Fröhner et al., 2019). Yet, another IBE-based study found that SoA over a robotic hand does not necessarily depend on the embodiment of the artificial limb (Caspar et al., 2015). Thus, we need to understand to what extent SoA is influenced by the effect of an external force on own motion.

In the present study, we varied the relevance of the force to the task-at-hand by providing the guiding force that can help or perturb the task performance of the user, and applied an IBE paradigm adapted for a physical HMI task to measure the perceptual bias resulted from the presence of the external guiding force. Specifically, the participants performed a reaching task to a target location using a force-feedback device and delayed visual feedback of a virtual cursor, a scenario that is likely to occur in teleoperation applications (Chopra et al., 2003). SoA was investigated using an adapting IBE paradigm in which the participants reported perceived delay of the own motion in the presence of the additional guiding force. We hypothesized IBE is sustained when guiding force adheres to a desired motion of the participants, while IBE is diminished when the force results in undesirable outcome.

## 2. Methods

### 2.1. Participants

Twenty-two participants took part in this study. The participants were healthy young adults (age = 25.0 ± 3.0 years old). Three were female, and all performed the task with their right hand. The study was conducted according to the Declaration of Helsinki, and all participants gave written informed consent before the participation. The study was approved by the research ethics committee of the Technical University of Munich (project no. 205/14).

### 2.2. Stimuli and Apparatus

**Figure 1:**
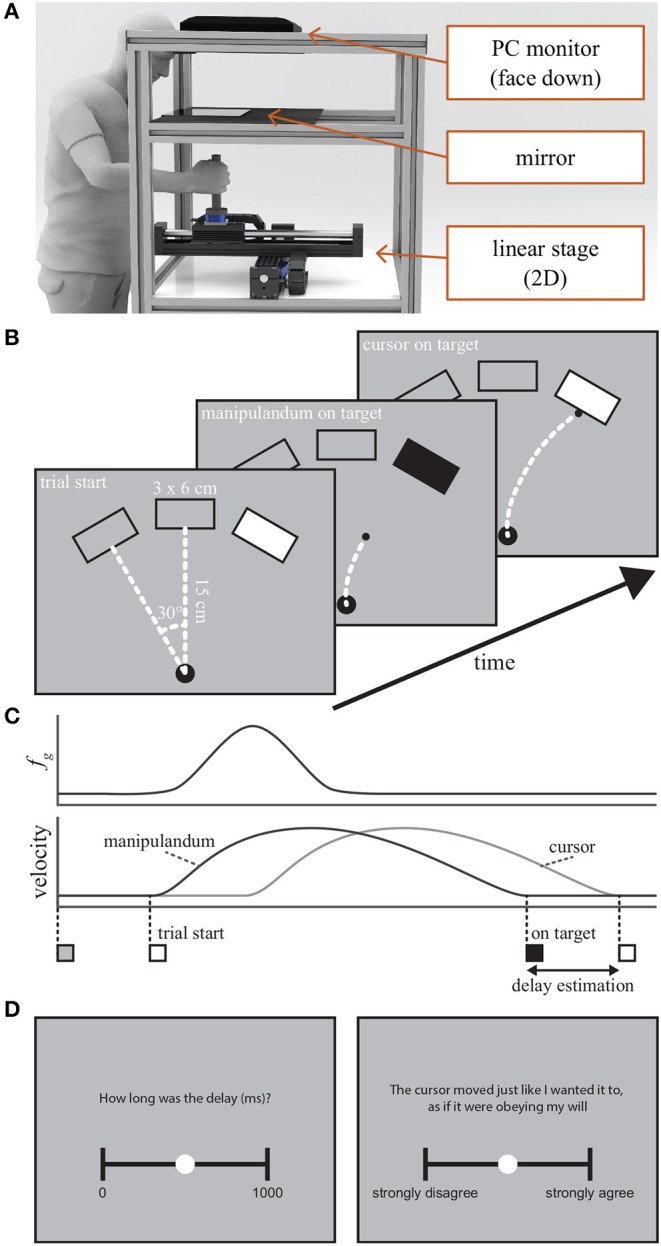
**(A)** An illustration of the workspace. The visual displayed was superimposed to the real workspace with a 2D linear stage manipulandum by mirroring the PC display. **(B)** The visual task space shown to the participants during the Reaching phase. The start of a trial was indicated by turning the color of the target platform to white. When the manipulandum and the delayed cursor reached the target, the platform changed the color to black and white, respectively. **(C)** An illustration of the manipulandum/cursor velocity profiles and guiding force (*f*_*g*_) in a single trial. **(D)** Scales for delay estimation and questionnaire.

During the experiment, the participants held a manipulandum with their right hand to control a cursor displayed on a computer screen. The visual position of the cursor and the targets were presented with a mirrored PC monitor. A 35 × 25 mm surface mirror glass (Screen-Tech, Germany) was horizontally placed 20 cm above the center of the manipulandum (Figure 1A). The PC monitor was placed face-down another 20 cm above the mirror. The location of the mirrored visual cursor was manually calibrated so it was aligned with the center of the handle position of the manipulandum as the participant's forehead was rested on the padded bar. The kinesthetic rendering is actualized by a Thrusttube Module (Copley Controls, USA). The actuation device consists of two sets of a single rail stage and a linear servo motor driven cart stacked perpendicularly to create a planar workspace. The device was positioned so the *x*-axis and *y*-axis of the workspace were respectively aligned with the mediolateral and sagittal axes of the participants. A vertical handle (1 cm radius) is mounted on the cart with a JR3-67M25 6-axis force/torque sensor (JR3 Inc., USA) between them in order to measure the force applied by a participant. The different stimuli were prepared for the reaching, delay estimation, and questionnaire phases as follows.

#### 2.2.1. Reaching Phase

The participants viewed a display showing a cursor, one starting platform, and three target platforms on a gray background (Figure 1B). The cursor was a black colored disk with 0.25 cm radius, and its motion was controlled by the participant using the manipulandum. The visualization of the cursor was delayed at one of three predefined latencies (see section 2.4) from the start of a trial until it reached the target, but otherwise the cursor position was aligned with the current manipulandum position, i.e., when returning to the starting platform. The starting platform was a 1 cm black radius circle placed midline at approximately 5 cm from the base of the screen. The cursor and the starting platform had the same color on purpose to prevent the participants from noticing when the visual cursor delay was introduced. The target platforms were a 3 × 6 cm gray colored rectangle with a black frame. One of them was placed on the midline so the front surface is 15 cm away from the starting platform and the remaining two platforms were rotated by ± 30 degrees at the same distance. The target for the reaching task was displayed by turning the color of one platform to white. When the manipulandum and the delayed cursor reached the target, the color changed into black and white, respectively. This allowed the participants only to focus on the period the target platform was colored black for the subsequent delay estimation phase.

#### 2.2.2. Delay Estimation and Questionnaire Phases

The delay estimation phase was used for registering the perceptual experience of the visual cursor delay similar to previous studies (Haggard et al., 2002; Caspar et al., 2015), and the questionnaire phase was used for obtaining self-reported SoA as a comparison to the objective IBE-based SoA. The manipulandum was locked during the entire phase and it was not movable. For both phases, a visual analog scale with a width of 15 cm was displayed at the center of the screen. For delay estimation, the continuous scale ranged from 0 to 1,000 ms without any intermediate points. For SoA questionnaire, the participants rated on the continuous scale of “strongly disagree” to “strongly agree.” For analyzing the self-reported SoA using the questionnaire, the agency items of the questionnaire from Caspar et al. (2015) were reformulated to fit our control-oriented task in the virtual environment. All items were displayed above the visual analog scale once at a time in a fixed order as shown in Table 1. The lateral force measurement of the manipulandum navigated the circle nozzle (0.5 radius), initially presented on the middle of the scale, to indicate the response by the participant. A forward push to the device above 10 N indicated a registration of the response.

**Table 1:** Questionnaire adapted from the SoA items from Caspar et al. (2015).

Item 1.	The cursor moved just like I wanted it to, as if it were obeying my will.
Item 2.	I felt as if I were controlling the movement of the cursor.
Item 3.	I felt as if I were causing the movement I saw.
Item 4.	Whenever I moved my hand, I expected the cursor to move in the same way.

### 2.3. Control Strategy of the Manipulandum

**Figure 2:**
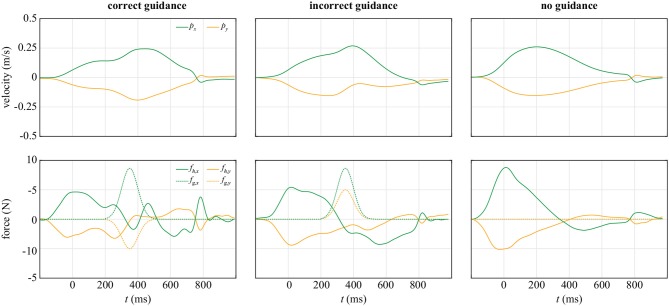
Exemplary velocities of the manipulandum and forces in a single trial. All examples are when the participants reached to the left target, and the incorrect guidance was directed toward the right target. The cursor fully left the starting circle at *t* = 0 which represented a start of a trial. The increase of *f*_*h*_ magnitude at around 800 ms is due to the contact with the virtual wall of the target platform.

The human operator interacts with the planar actuation device by grasping the handle to move the cursor in virtual reality, as depicted in Figure 1. The manipulandum is controlled to have a simulated mass of 5 kg. During the experiment, a guiding force was applied to the manipulandum, with the force profile of a normal distribution curve with 10 N peak force spanned over 300 ms pushing to the target platform surface after 200 ms from the movement onset. Exemplary velocities of the device and forces applied by the human, as well as the guidance forces are depicted in Figure 2 for the correct, incorrect, and no guidance, respectively. For more details on control of the manipulandum, see Appendix.

### 2.4. Design

We used a 3 × 3 within-subject design, consisting of directional correctness of the guiding force and delay of the visual cursor motion as the independent variables. The first independent variable was the Guiding force to the manipulandum either in the correct or incorrect directions, or being absent entirely, i.e., “no force.” In the correct force condition, the guiding force was always directed to the illuminated target. For the incorrect force condition, the force was pseudo-randomly directed to one of the two non-target directions. In the no force condition, no guiding force was applied. Each force condition was presented as a block of trials in random order across the participants. The target location as well as the non-target force direction were varied every trial so the probability of each event was balanced. The second independent variable was the Delay of the visual feedback for which the visual cursor was delayed by either [300, 500, 700] ms. The participants were informed about the delay prior to the experiment, but were told the latency would have been randomly chosen between 0 and 1,000 ms. The participants completed 135 trials for three blocks, totaling 405 trial for the whole experiment. When the participants failed to reach the correct target, the same trial was repeated at the end of the block in the order of failed trails. The total error rate was 2.7%.

In order to investigate the change in IBE according to the Guiding force, the deviation of the perceived delay from the real delay was calculated, and this perceptual bias was used as a dependent variable for statistical analysis using a 3 (Guiding force) × 3 (Delay) repeated-measures ANOVA. In order to supplement the objective SoA results in terms of IBE, the questionnaire administered at the end of each block addressed the subjective SoA with regard to the force feedback type using the analog scale. Thus, the self-reported SoA refers only to the Guiding force and the experience over the Delay conditions was pooled together. The agreements to SoA from the four items were normalized between 0 (strongly disagree) − 1 (strongly agree), and averaged to derive the subjective SoA for statistical analysis using a one-way repeated-measures ANOVA. *Post-hoc* analyses were carried out with the Bonferroni correction. The alpha value was set to 0.05 for statistical significance. In order to explore the relationship between the self-reported SoA and the time perception bias, the Pearson's correlation was calculated between the questionnaire scores and the perceptual bias. In the experiment, the questionnaire was only administered at the end of each guiding force block, and it reflected experiences over all three visual delays. Thus, the means of the perceptual bias in each guiding force type were calculated to make the variable comparable to the questionnaire scores. The individual differences in the two variables were minimized by performing the z-transformation for data from each participant, resulting in 3 (guiding forces) × 22 (participants) samples. Furthermore, in order to evaluate how the different guiding force types influenced the reaching performance, we analyzed the trial duration, peak interaction force and the manipulation share. As defined in Donner et al. (2018), the interaction force is calculated as

(1) $$\begin{array}{l} f_{int}=\frac{1}{2}\mathrm{sgn}\left(f_{h}\right)\left(\left|f_{h}|+|f_{g}|-|f_{h}+f_{g}\right|\right), \end{array}$$

wherein *f*_*h*_ and *f*_*g*_ are the forces applied by the human and the guiding force, respectively. Namely, the interaction force represents the force that is compensated by the two agents and has no contribution to the motion of the handle. Thus, excessively high interaction force suggests inefficiency in coordination between the agents, although the interaction force may become a source of communication between agents (Donner et al., 2018). The largest interaction force during a trial was then sampled for statistical analysis as an index of (in)efficiency in interaction. The manipulation force was the non-compensated force which resulted in motion of the cursor,

(2) $$\begin{array}{l} f_{mnp}=f_{h}-f_{int}. \end{array}$$

The manipulation share is calculated by normalizing *f*_*mnp*_ to the total manipulation force working on the manipulandum, which gives percentage of the participants' force resulted in cursor motion, or dominance in task execution. As *f*_*int*_ and *f*_*mnp*_ respectively result in 0 N and 100 % in the no guiding force condition, 2 (Guiding force) × 3 (Delay) ANOVAs were used for statistical analysis without the no guiding force condition. Alpha-level of 0.05 was used for statistical significance of all tests.

### 2.5. Procedure

Participants stood in front of the manipulandum to perform the task with the right arm. The chair height was adjusted so the manipulandum handle came slightly above the waist. At the beginning of each trial, the participants held the handle and moved the cursor to the start position by looking into the mirrored display. At this stage, the cursor was not delayed. One second after the cursor was placed on the starting platform, the trial commenced by coloring one of the target platforms. The participants were instructed to complete the task by moving the handle to the white-colored target at a comfortable speed. When the handle arrived at the target, the color changed to black. The participant was then asked to hold the cursor at the target until the delayed cursor arrived to the target, and saw the target color changed back to white. The participants were told to focus on the period the target platform was colored black for the subsequent delay estimation test. After 1 s lapse, the handle was locked to prevent from further motion and a visual display on the screen asked the participant to rate the cursor delay on the visual analog scale. The participant then moved the cursor on the scale by laterally applying force to the handle in order to report their opinion. A forward push by the participant indicated the completion of the rating. When the response was registered, the handle lock was removed and the participant freely moved the handle to the start platform for the next trial. After completion of each experimental block, participants filled in the agency questionnaire with the same procedure as the delay estimation for the four questionnaire items.

Our pilot study indicated that participants found challenging to estimate the time-lapse in less than 1,000 ms time-frame. Thus, a familiarization block was prepared in which all target boxes flashed simultaneously from white to black, held it for a reference time of [200, 400, 600, 800] ms, and then to white again. The procedure was repeated 10 times for each reference time with an inter-stimulus interval of 2 s. The presentation order was counterbalanced between the participants in an increasing or a decreasing order. The reference time was also displayed at the center of the screen to help the participants to learn and calibrate visual time perception. Furthermore, the participants were allowed to practice the task with a random visual cursor delay between 0 and 1,000 ms without external force until they felt comfortable with proceeding with the main experiment after the familiarization block.

## 3. Results

### 3.1. Intentional Binding Effect

**Figure 3:**
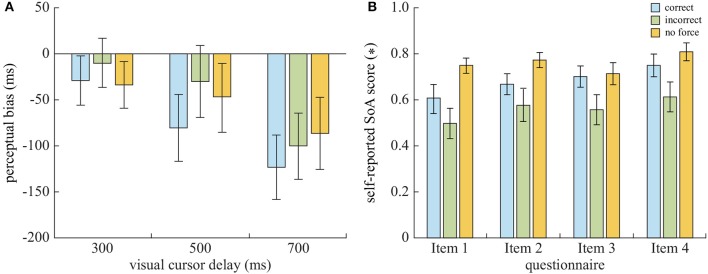
**(A)** The perceptual bias in the cursor delay estimation. In general, the participants estimated the delay shorter than the actual. **(B)** The qualitative SoA measured from the four-item questionnaire. The error bars represent one standard error.

The analysis revealed the participants overall perceived the delay to be rather shorter than the actual visual delay (−60.2 ± 88.9 ms, see Figure 3). This tendency was stronger with increasing visual delays, as the perceptual delay was the smallest when estimating the 300 ms delay (−24.3±85.8 ms) and the largest with 700 ms delay (−103.4±105.6 ms). The 3 x 3 repeated-measures ANOVA confirmed a main effect of the Delay in the biased delay perception, *F*_(2, 42)_ = 11.12, *p* < 0.0005, partial η^2^ = 0.35. The *post-hoc* analysis confirmed the significant differences between 300 vs. 700 ms (*p* < 0.01), as well as 500 vs. 700 ms (*p* < 0.001), but not 300 vs. 500 ms (*p* = 0.31). Although there was no main effect of the Guiding force (*p* = 0.115), there was an interaction effect between the Guiding force and Delay, *F*_(4, 84)_ = 2.71, *p* < 0.05, partial η^2^ = 0.11. When the Guiding force was in the correct direction or no force feedback was given, the time-lapse between the two events was perceived as shorter (−77.6±105.8 ms and −56.0±94.9 ms, respectively) than for the force acting in the incorrect direction (−46.9±90.8 ms). This IBE-like observation was evident for estimating 300 and 500 ms delays, but not for 700 ms delay estimation.

### 3.2. Self-Reported SoA and Correlation to the Perceptual Bias

**Figure 4:**
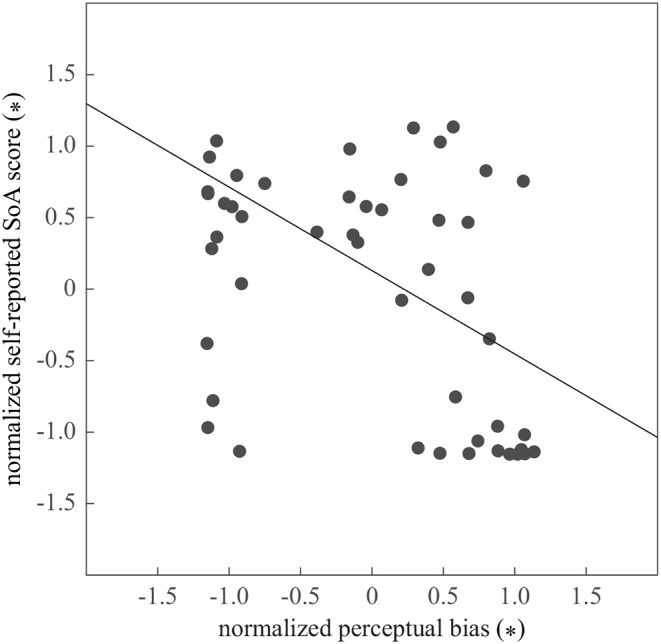
The relationship between the bias in perceiving visual delay and self-reported SoA. The variables were z-transformed to normalize across participants. The line shows the linear regression.

In accordance with the results of the delay perception bias, the self-reported SoA was lower with the incorrect guiding force (0.56±0.26) than with the correct guiding force (0.68±0.19) and with no guiding force (0.76±0.13). The repeated-measures ANOVA on the four questionnaire items revealed a statistically significant difference in delay estimation due to the Guiding force, *F*_(2, 42)_ = 9.62, *p* < 0.0005, partial η^2^ = 0.31. The *post-hoc* analysis revealed the difference between the incorrect and no guiding forces was significant (*p* < 0.005), and there was a trend of difference between the correct and incorrect forces (*p* < 0.07). Furthermore, mild correlation of the self-reported SoA and the bias in time perception was found, *r* = −0.41, *p* < 0.001, supporting the negative relationship between self-reported SoA and the perception of the visual event delay (Figure 4).

### 3.3. Performance Measures

**Figure 5:**
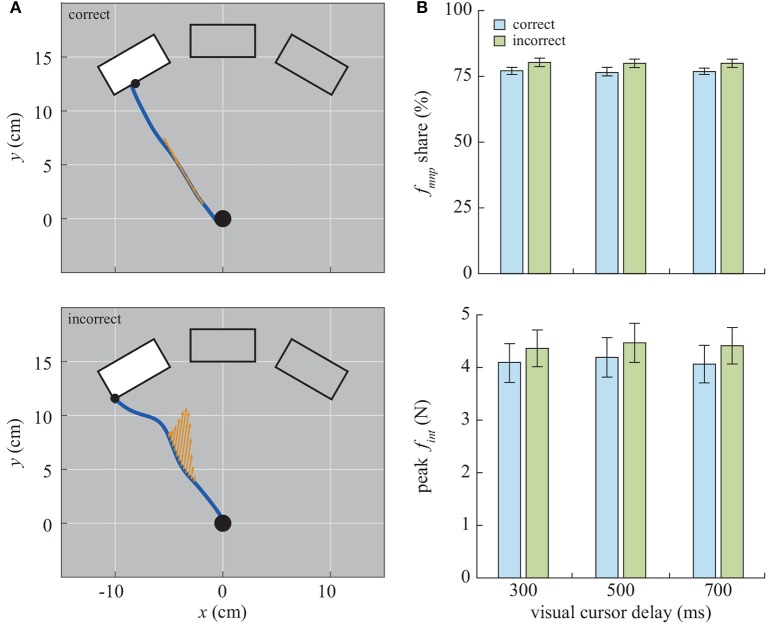
**(A)** An illustration of the correct and incorrect force vectors from a single trial. The angle and the length of the arrows represent the magnitude and the direction of the guiding force, respectively. **(B)** The averaged manipulation share of the participants and the peak interaction force. The data from no-force condition is omitted.

On average, the participants spent 543.0±175.4 ms to complete a single trial and our experimental manipulation did not influence the trial duration, as indicated by the ANOVA (*ps* > 0.24). Furthermore, the observed peak interaction force during the trial was (5.17±1.85*N*, see Figure 5), and the 2 × 3 ANOVA indicated neither the Guiding force (*p* = 0.31) nor the Delay (*p* = 0.10) influenced the interaction force. The analysis on the manipulation share revealed that the participants were responsible for manipulating the cursor by 77.4±6.2 % with the correct guiding force, while the manipulation share was higher with the incorrect guiding force (80.3±4.6 %) due to a lack of assistance. The ANOVA confirmed the main effect of the Guiding force on the manipulation share [*F*_(1, 21)_ = 5.06, *p* < 0.04, partial η^2^ = 0.19]. The *post-hoc* analysis confirmed the manipulation share difference between the correct and incorrect guiding force (*p* < 0.001). On the other hand no main effect of Delay (*p* = 0.33) or the interaction effect (*p* = 0.68) was found.

## 4. Discussions

The present experiment investigated whether SoA about voluntary movement is modulated in the presence of external, independent forces effecting the task execution in physical HMI. Given the observation that people experience SoA collectively when a set of actions performed by more than one individual is assimilated (Dewey and Knoblich, 2014), we hypothesized that the external force may be attributed as own when the guiding force lead to a desired outcome. Our results confirmed this hypothesis; the perceptual bias in delay estimation was least when the guiding force led to a wrong target, indicating the less SoA compared to when the guiding force was assistive or when the entire motion was performed by the participants, i.e., in the condition without providing a guiding force. The observed IBE was around 50−80 ms which is in accordance with previous studies (e.g., Caspar et al., 2015). Furthermore, the results are consistent with the questionnaire-based self-reported SoA score which also indicated lower but sustained SoA when the assistive force was supporting rather than perturbing. One exception was that our SoA scores seems to have been influenced by the magnitude of the event delay , and IBE was not observable for the delay of 700 ms. Remarkably, the participants reported stronger agency when being correctly assisted, while their actual contribution to the action, i.e., their manipulation share, was actually lower than with the perturbing controller. It is, however, important to note our questionnaire consisted of only positive worded statements in the ascending Likert-scale due to experimental time constraints. Thus, the results may have been subject tothe extreme and acquiescence bias (Nunnally and Bernstein, 1994) that could have exaggerated the overall agreement magnitude in their responses. In addition, the correlational analysis between the self-reported SoA and the delay perception bias only revealed a mild relationship. This may be partly due to the fact that our questionnaire scores were affected by the response bias. The supplementary analysis showed the correct guiding force lowered the amount of force required by the participants to move the manipulandum to the target. Thus, the participants contributed less to the task execution in this condition. In contrast, the guiding force did not influence the performance of the task in terms of the trial duration or the interaction force. Thus, lowered physical work load with the assistive controller appears not to reduce SoA, but the characteristics of the external force may have influenced our SoA scores.

Literature suggests that comparator models (Wolpert et al., 1995; Miall and Wolpert, 1996) play an important role in identifying the discrepancy between the intended movements and the sensory outcome (Spengler et al., 2009). Consistently, SoA is reduced when the discrepancy such as spatial misalignment or temporal delay is introduced. For instance, past studies (Sato and Yasuda, 2005; Farrer et al., 2008) observed agency can be misattributed to an external source when the outcome of an action is incongruent with own predicted sensory outcome, and attributing the motion to an external source. In reverse, the central nervous system can distinguish self-action and external sources through learning (Synofzik et al., 2006; Novembre et al., 2012; Pesquita et al., 2017). The monitoring of the sensory-motor error in regard with expected outcome may thus be linked to SoA (Bellebaum et al., 2010). However, a question remains how and to what extent assisting/perturbing external force influences SoA. In order to ensure that the guiding force can be rated as assistive or perturbing, we employed a simple decision-making task in the present study. Although the direction of the incorrect force was varied, there were only three possibilities and the predictability was relatively high. Nevertheless, the perceptual delay bias was not affected and it seems that the mere predictability does not play a role in increasing SoA. A similar observation was reported by the work of Desantis et al. (2012), which concluded prior causal beliefs about the action rather than the predictability of it was an important factor for SoA. In our study, therefore, the high compatibility of the guiding force and the desired action outcomes may have yielded (mis-)attribution of the observed motion as own action. However, we do not know how SoA correlates with undesired external force as our study employed a simple decision-making task. Thus, we need to explore how the action error is processed in the central nervous system and the self/other action attribution is performed.

Our results indicate that the desirable guidance force helped the participants to establish an experience of collective agency with an external device in our task. However, the experimental power was found to be considerably small, and was absent in longer inter-stimulus-intervals. We believe that the main reason for this is that it had been overwritten by other sources of sensory-motor biases. At larger time-intervals, for example, uncertainty about the sensory-motor events arises and may shadow other effects. In support of this claim„ research has shown reduced sensitivity to a stimulus duration of longer inter-stimulus-intervals in various perceptual (Plomp et al., 2012) and motor coordination tasks (Wing and Kristofferson, 1973). In addition, the correlational analysis indicated the relationship between the perceptual bias and the self-reported SoA is only mild, although our observation is likely to be confounded by the inclusion of the cases with the long-inter-stimulus-interval in the analysis. Similarly, the time-frame of the response between the two measures were different as the questionnaire was the end-summary of 135 trials, while the perceptual bias was measured immediately after each trial. Thus, interpretation of the observed perceptual delay against self-reported SoA should be exercised cautiously. Another issue in using SoA scores for evaluating and differentiating more complex controllers is the fact that we have to delay the visual feedback in our paradigm. For a control design aiming at optimizing user experience, we would need to estimate SoA ambiently so the controller can adapt online, e.g., using cognitive models (Schürmann et al., 2019). For instance, controlling the attitude of the robot toward disagreements in early phases of the interaction is considered as an important issue for HMI (Hancock et al., 2011). Therefore, perturbing the task by deferring the visual feedback is not a desirable option for many applications. Although our experimental design used an artificial setting of physical HMI, the present study successfully demonstrated the relevance of SoA in physical HMI using a controlled and a well-validated methodology. Thus, methodological improvements to monitor SoA in realistic HMI such prosthetic will be an important next step. While we need more research into how effectively we could extract the perceptual bias caused by changes in SoA, the current paradigm can be used to evaluate how external forces are (mis-)attributed as own motions in a control design. Having control over action is the important cue for sustaining SoA (Beck et al., 2017), and our study shows that the sense of control is at least partially independent of the physical effort the individual had contributed to the task. Instead, the congruency between the movement and the desired outcome is a crucial factor. Despite the variety of interesting effects and the increasing importance of autonomous agents, the interaction of those with human users is not yet fully understood. While the underlying mechanisms causing the perceptual bias observed in the intentional binding paradigm are still to be understood, we believe that this study distinctly contributes to the understanding of how a control design in physical HMI modulates SoA.

## 5. Conclusion

This study used an adapted intentional binding effect (IBE) paradigm to investigate whether SoA can be used to measure the quality and experience of physical HMI schemes that allow the human operator and the collaborative machine to act as a “single entity.” Our study demonstrated that motion caused by an external force can be attributed to own cause when it results in a desired outcome. Furthermore, the study indicates IBE may be useful for objectively evaluating a controller, although the experimental power is considerably small and might be influenced by various other factors. Moreover, we observed IBE results to be consistent with the self-reported SoA scores from the questionnaire report. Interestingly, assistance seems to improve IBE despite being supported by another agent. Advancing the understanding of IBE will help us to isolate the true perceptual bias resulted from SoA, and extension of the paradigm to unperturbed real use cases will be essential for an adaptive user-centric HMI scheme.

## Data Availability Statement

The data set analyses for this study can be downloaded from https://syncandshare.lrz.de/getlink/fiFQhwTh5EbVWJg637bzoAHt/frontier_endo_2020.zip.

## Ethics Statement

The studies involving human participants were reviewed and approved by Technical University of Munich, Arcisstraße 21, 80333 München, Germany. The patients/participants provided their written informed consent to participate in this study.

## Author Contributions

SE and PB developed the idea. JF executed the study. SE, JF, and SM drafted the manuscript. SH and PB equally provided critical revisions to the draft. All authors contributed equally to its conception.

## Conflict of Interest

The authors declare that the research was conducted in the absence of any commercial or financial relationships that could be construed as a potential conflict of interest.

## Appendix

### Control Strategy of the Manipulandum

The manipulandum has two translational degrees of freedom in the *x* − *y* plane. The *admittance controller* is used to enforce *compliant behavior* of the planar device at the interaction point with the physically coupled human. Desired dynamics of the device, achieved with the admittance controller, is defined as a virtual mass-damper system

(A1) $$\begin{array}{l} M\overset{¨}{p}+D\overset{˙}{p}=f_{h}+f_{ff}, \end{array}$$

where $M={\left[5\;5\right]}^{⊤}kg$ and $D={\left[15\;15\right]}^{⊤}Ns/m$ are the desired inertia and damping matrices of the device, respectively. The position of the handle is $p={\left[p_{x}\;p_{y}\right]}^{\text{T}}$. The acceleration and velocity vectors are denoted as $\ddot{p}$ and $\dot{p}$, respectively. The force exerted on the handle by the human is denoted as the vector ***f***_*h*_. The feed-forward force ***f***_*ff*_, is composed of the *guiding force* ***f***_*g*_, exhibited at the start of the motion, and *target assistive force* ***f***_*t*_, exhibited when the cursor collides with the target, given

$$\begin{array}{l} f_{ff}=f_{g}+f_{t}. \end{array}$$

The guiding force, ***f***_*g*_, is defined as a normal distribution curve with 10 N peak force spanned over 300 ms pushing in the direction of the middle of the target platform surface. It occurred 200 ms after the handle moved out of the starting platform and was enough to push the cart to the target without applying any additional force by the human if the handle motion was not resisted by the human, such that

(A2) $$\begin{array}{l} f_{g}(t)=\{\begin{array}{ll} 10{\text{e}}^{-\frac{{\left(t-350\right)}^{2}}{4000}}, & \text{if }200\leq t\leq 500\text{ ms and }||p|{|}^{2}\geq 1\text{ cm}\\ 0, & \text{otherwise} \end{array} \end{array}$$

The target assistive force component, ***f***_*t*_, is enforced once the target is reached, by applying *f*_*t*_ = 20 N to the handle. The desired dynamics given with (A1) are achieved with a proportional-derivative controller. The controller is implemented in *Matlab/SIMULINK*. It runs on a computer with *Linux PREEMPT real-time kernel* (Ubuntu 14.04, 3.14.3-rt4) with a fixed-step solver at the sampling rate of 1 kHz.
